# The complete *Ac/Ds *transposon family of maize

**DOI:** 10.1186/1471-2164-12-588

**Published:** 2011-12-01

**Authors:** Chunguang Du, Andrew Hoffman, Limei He, Jason Caronna, Hugo K Dooner

**Affiliations:** 1 Dept. of Biology & Molecular Biology, Montclair State University, Montclair, NJ 07043, USA; 2 Waksman Institute, Rutgers University, Piscataway, NJ 08854, USA; 3 Dept. of Plant Biology, Rutgers University, New Brunswick, NJ 08901, USA

## Abstract

**Background:**

The nonautonomous maize *Ds *transposons can only move in the presence of the autonomous element *Ac*. They comprise a heterogeneous group that share 11-bp terminal inverted repeats (TIRs) and some subterminal repeats, but vary greatly in size and composition. Three classes of *Ds *elements can cause mutations: *Ds-del*, internal deletions of the 4.6-kb *Ac *element; *Ds1*, ~400-bp in size and sharing little homology with *Ac*, and *Ds2*, variably-sized elements containing about 0.5 kb from the *Ac *termini and unrelated internal sequences. Here, we analyze the entire complement of *Ds*-related sequences in the genome of the inbred B73 and ask whether additional classes of *Ds*-like (*Ds-l*) elements, not uncovered genetically, are mobilized by *Ac*. We also compare the makeup of *Ds*-related sequences in two maize inbreds of different origin.

**Results:**

We found 903 elements with 11-bp *Ac*/*Ds *TIRs flanked by 8-bp target site duplications. Three resemble *Ac*, but carry small rearrangements. The others are much shorter, once extraneous insertions are removed. There are 331 *Ds1 *and 39 *Ds2 *elements, many of which are likely mobilized by *Ac*, and two novel classes of *Ds-l *elements. *Ds-l3 *elements lack subterminal homology with *Ac*, but carry transposase gene fragments, and represent decaying *Ac *elements. There are 44 such elements in B73. *Ds-l4 *elements share little similarity with *Ac *outside of the 11-bp TIR, have a modal length of ~1 kb, and carry filler DNA which, in a few cases, could be matched to gene fragments. Most *Ds*-related elements in B73 (486/903) fall in this class. None of the *Ds-l *elements tested responded to *Ac*. Only half of *Ds *insertion sites examined are shared between the inbreds B73 and W22.

**Conclusions:**

The majority of *Ds*-related sequences in maize correspond to *Ds-l *elements that do not transpose in the presence of *Ac*. Unlike actively transposing elements, many *Ds-l *elements are inserted in repetitive DNA, where they probably become methylated and begin to decay. The filler DNA present in most elements is occasionally captured from genes, a rare feature in transposons of the *hAT *superfamily to which *Ds *belongs. Maize inbreds of different origin are highly polymorphic in their DNA transposon makeup.

## Background

The first transposable element discovered by McClintock [1] was *Ds *(*Dissociation*). *Ds *could break chromosomes at its site of insertion and could move in response to another factor, which she named *Ac *(*Activator*) and showed to be self-mobilizing or autonomous [2]. Shortly after this discovery, McClintock established that there were two types of *Ac*-responsive *Ds *elements: those that caused chromosome breaks at high frequency and those that did not. She named the former state I *Ds *and the latter, state II *Ds *[3]. Thus, early on, it became clear that *Ds *elements could differ genetically. Yet, a common origin for these elements was suggested by the observation that state I elements could change to state II. How heterogeneous *Ds *elements are only became clear after *Ac *and *Ds *were isolated and characterized molecularly [4-6].

The 4.6-kb autonomous *Ac *element makes a single 3.8-kb transcript that spans most of the element's length and encodes an 807-amino acid putative transposase [7]. It causes an 8-bp target site duplication (TSD), ends in 11-bp terminal inverted repeats (TIRs), and contains, within the terminal 200 bp at either end, multiple copies of a hexameric repeat to which the *Ac *transposase binds [8]. *Ds *elements share TIRs with *Ac *and also cause 8-bp duplications of the target site. Three very different kinds of *Ds *elements can transpose and cause mutations in response to *Ac*. *Ds-del *elements are simple internal deletion derivatives of *Ac *found predominantly in genetic stocks that recently carried an active *Ac *[4,9,10]. *Ds1 *elements are short (~400 bp) and share with *Ac *only the 11 bp TIRs and a few of the subterminal hexameric repeats [11]. *Ds2 *elements are > 1 kb in length and share extensive sequence homology with *Ac *in the terminal 200 bp at either end [12]. In many *Ds2 *elements, a large part of *Ac*'s internal sequence has been replaced with an unrelated or "filler" sequence. The ability of *Ds *elements to cause chromosome breaks turned out to be a function of their structure: all chromosome breakers have multiple transposon ends [5,13,14].

*Ac *and genetically defined *Ds *elements transpose preferentially into unique or low-copy sequences and largely avoid the repetitive DNA that makes up the bulk of the maize genome [15,16]. Therefore, these elements are highly efficient insertion mutagens. *Ac *is absent from most maize lines or populations and present in usually one copy in lines with *Ac *activity. In contrast, many *Ds-*hybridizing sequences are present in the genomes of all lines examined [17], but there has not been yet a concerted effort to characterize all the sequences related to *Ds *in the maize genome. To that end, we created a heuristic searching algorithm based on the sequence of the *Ac*/*Ds *TIRs and the size of the TSD and ran it through the maize pseudomolecules. Each *Ds*-related element identified via this search was individually numbered, annotated, and had its insertion site categorized according to uniqueness and content in the B73 maize genome sequence [18].

A total of 903 elements with *Ds *sequence features were located within the B73 genome. A minority resembled previously described elements: there are 331 *Ds1 *and 39 *Ds2 *elements, most of which are probably mobilized by *Ac*. In addition, two new classes of *Ds-like (Ds-l) *elements were identified. *Ds-l3 *elements lack extensive subterminal homology with *Ac*, but carry fragments from various parts of the *Ac *transposase gene. There are 44 such elements in B73. *Ds-l4 *elements share little similarity with *Ac *outside of the 11-bp TIR and have a modal length of ~1 kb. The majority of *Ds*-related elements in B73 (486/903) fall in this class. None of the *Ds-l *elements tested were excised by *Ac*. Unlike *Ac *and recently transposed *Ds *elements, about half of the *Ds*-related elements identified in this study had inserted in repetitive DNA. Conversely, repetitive sequences, such as long terminal repeat (LTR) retrotransposons, were found within some *Ds-l *elements. Some elements carried gene fragments, a rare feature of transposons of the *hAT *superfamily to which *Ds *belongs, though a common feature of other transposons. Lastly, only half of *Ds *insertion sites examined were shared between the inbreds B73 and W22, indicating that the makeup of DNA transposons will vary greatly among inbreds of unrelated origin.

## Methods

### Development and implementation of the *Ds *discovery algorithm

The 11-bp terminal inverted repeats (TIR) are specific sequences that define the 5' end (C/TAGGGATGAAA) and 3' end (TTTCATCCCTA) of each *Ac/Ds *element and play a key role in transposition [19-22]. The other identifying trait of an *Ac/Ds *element is the target site duplication (TSD). Not part of the transposon, the TSD is a direct repeat of the same 8 base pairs upstream and downstream of the TIR. Unlike the 11-bp TIR, the 8-bp TSD is not a specific sequence. Rather, because it is the site where the *Ds *element inserted into the genome, the TSD can be almost any combination of 8 bp. Based on the sequence characteristics of *Ac *and *Ds *elements, we developed a data mining algorithm, written in PERL, to search through the maize pseudomolecules for *Ds *sequences and found sequences with perfect TIRs and identical TSD sequences and with TIRs and TSD sequences differing by up to 2 bp. The algorithm generated putative *Ds *elements and identified the position of their TSD in the genome. Putative *Ds *elements were then used to BLAST-search the maize genome database for related *Ds *sequences in which the TIRs or TSDs differed by more than 2 bp.

Because known *Ds1 *elements are smaller than 500 bp and *Ds2 *elements are larger than 1 kb, all elements were first separated by length into groups of elements measuring < 500 bp, 500 to 1000 bp, 1000 to 5000 bp, and > 5000 bp. Then, elements were located in the maize genome via BLAST, and redundant sequences were eliminated. Each sequence was then compared to known *Ds1 *and *Ds2 *elements using BLAST2, and categorized based on those results. The nature of the target sites was determined by a series of tests, including presence of start and stop codons, presence of untranslated region sequences, EST (expressed sequence tag) support, and presence of corresponding mRNA in GenBank.

Prior annotation of the predicted *Ds *elements and of the genome sequences 200 bp upstream and downstream from the element's insertion site were checked in the database at maizesequence.org. The 200 bp flanking each element were analyzed for uniqueness using BLAST, maizesequence.org, and RepeatMasker http://www.repeatmasker.org/. The 200-bp subterminal regions within each sequence were examined for the AAACGG 6-bp hexamer repeats which bind the *Ac *transposase [8]. Only three copies of these hexamers in the 5' end have been shown to be required for transposition [23].

### Assay for *Ds *mobility

The B73 stock used in the *Ds *mobility assay was obtained from the USDA North Central Region Plant Introduction Station at Ames, IA (PI-550473). The W22 stocks used were: the standard *Wx *version and a version carrying *wx-m7(Ac)*, an unstable *wx *allele described by McClintock [24]. This allele arose by insertion of the 4.6-kb *Ac *element in the 5' untranslated region of the *Wx *gene [20,25]. Activation of *Ds *excision by *Ac *was monitored by PCR in an F1 between B73 and the W22 stock carrying *wx-m7(Ac) *[15]. DNA was extracted from young seedling leaves and PCR reactions were run as described [26]. PCR primers were designed based on the 500 bp sequences flanking each *Ds *element end. All primer sequences are listed in Additional File 1, Table S1.

### Analysis of *Ds *elements in W22

Physically sheared W22 DNA was size-fractionated by agarose gel electrophoresis to a 300- to 500-bp size range. Sheared DNA ends were blunt-ended and kinased with an Epicentre End-It DNA end repairing kit and adaptors were ligated with T4 DNA ligase. PCR amplifications were carried out with an adaptor primer and primers ending in the *Ac/Ds *TIRs. PCR products were cloned into pGEM-T easy vector and sequenced in a 3730 DNA analyzer.

## Results

### Structure of *Ds*-related elements

**Figure 1:**
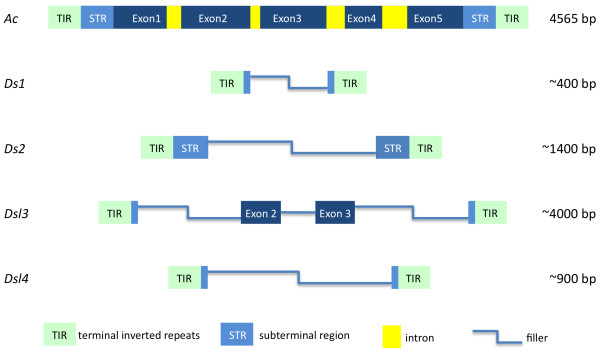
**Structure of different types of *Ds *elements compared with *Ac***. *Ac *is 4565-bp long and encodes a 5-exon transposase. *Ds1 *elements are the shortest and share little in common with *Ac*. *Ds2 *elements have ~200 bp of the *Ac *subterminal region at each end. *Ds-l3 *elements carry sequences corresponding to parts of exons 2 and 3 of the *Ac *transposase and are the longest *Ds *elements, on average. *Ds-l4 *elements have a modal length of about 1 kb and share with *Ac *only about 30 bp at either end.

*Ds1 *elements are short, less than 500 bp in length, and share little sequence in common with *Ac*, other than the 11-bp TIRs and a few copies of the AAACGG hexameric repeat found in the *Ac *subterminal regions (Figure 1). They were first detected in unstable mutations [6,27,28] and are known to occur in at least 50 copies in the genomes of all maize lines [29]. In contrast, *Ds2 *elements are much closer to *Ac*, sharing with *Ac *the ~200 bp subterminal regions (STR) and variable stretches of internal sequence, and appear to have originated from *Ac *by deletion (Figure 1). Most carry, in addition, "filler" sequence from other parts of the genome [30,31]. They have been estimated to occur in about a dozen copies and, like *Ds1*, have been found in several unstable mutations.

In addition to the previously described *Ds1 *and *Ds2 *element classes, we have identified a large number of elements that, though containing the hallmark *Ac*/*Ds *TIRs and flanking an 8-bp duplication, do not match any previously described *Ds *sequences. They lack obvious homology to *Ac *in the terminal 200 bp sequences and may not be mobilized by *Ac*, so we have referred to them as *Ds-like *(*Ds-l*) elements. *Ds-l *elements can be divided into two groups, *Ds-l3 *and *Ds-l4*. *Ds-l3 *elements have an average length of 4 kb and retain parts of exons 2 and 3 of the *Ac *transposase (Figure 1). *Ds-l4 *elements are on average about 1 kb long and, though very numerous and variable in sequence, do not differ much in size. The homology between *Ds-l4 *and *Ac *is restricted to little more than 30 bp on the 5' and 3' ends (Figure 1).

### Content of *Ds*-related elements in B73

**Figure 2:**
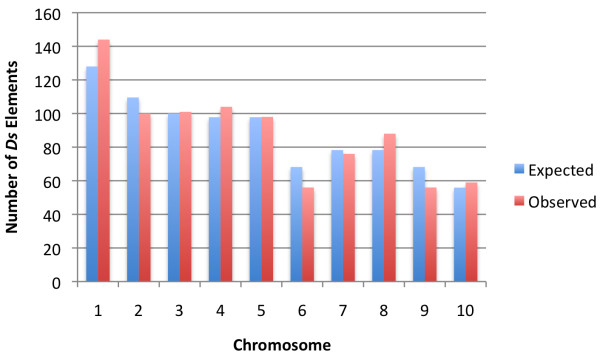
**Distribution of *Ds *elements in the 10 chromosomes of B73**. The observed distributions are not significantly different from those expected based on the length of the 10 chromosomes (χ^2 ^= 9.05, 9 df, 0.5 > P > 0.3).

The chromosomal distribution of all 903 *Ds*-related elements in B73 is shown in Table 1. There are 331 *Ds1*, 39 *Ds2*, 44 *Ds-l3*, and 486 *Ds-l4 *elements in the B73 genome (Additional File 1, Tables S2-S5). A χ^2 ^test showed no significant difference between the observed distribution of *Ds-*related elements per chromosome and the expected distribution based on chromosome length in the 10 chromosomes of B73 (Figure 2).

**Table 1:** Chromosomal distribution of *Ds *elements in B73

Chromosome	*Ds1*	*Ds2*	*Ds-l3*	*Ds-l4*	*Ac-like*	Total
1	48	7	4	84	1	144
2	45	5	3	46	1	100
3	40	2	6	53	0	101
4	28	2	5	69	0	104
5	42	4	4	47	1	98
6	20	0	3	33	0	56
7	26	3	5	42	0	76
8	31	6	8	43	0	88
9	26	2	0	28	0	56
10	17	4	3	35	0	59
Unknown	8	4	3	6	0	21

Total	331	39	44	486	3	903

Three elements, classified here as *Ac*-like elements, share a large amount of similarity with *Ac*, save for small indels. A similar element called *Ac-cryptic *has been described previously [32]. As anticipated from its history [33], the B73 genome contains no simple deletion derivatives of *Ac*. More than 90% of *Ds*-related elements in the genome fall either in the *Ds1 *or *Ds-l4 *classes. *Ds1 *elements were known to be numerous based on earlier hybridization data [11], but their copy number had been estimated to be around 50, so the present analysis of the maize genome sequence pushes up their number at least sixfold. Of the 331 *Ds1 *elements in B73, 184 were identified as such by the maize sequencing effort (maizesequence.org), 4 were incompletely annotated, and 143 were missed (Table 2). All 39 *Ds2 *elements identified here had variable filler sequences unrelated to *Ac*. Of the 39, only 2 were fully annotated as *Ds *sequences in the maizesequence.org database, 26 were partially annotated, and 11 were missed. We searched the B73 genome for the specific *Ds2 *elements previously isolated from unstable mutations and found that the element in *wx-B4 *[34] was present in two copies (*Ds2-2 *and *Ds2-15 *in Additional File 1, Table S3), but those in *adh1-2F11 *[12] or *sh2-m1 *[30] were absent.

**Table 2:** New and previously identified *Ds *elements in B73

		Previously Identified		
			
Element	New	Partially annotated	Fully annotated	Total
*Ds1*	143	4	184	331
*Ds2*	11	26	2	39
*Ds-l3*	2	42	0	44
*Ds-l4*	424	62	0	486
*Ac-like*	0	3	0	3

Total	580	137	186	903

None of the *Ds-l *elements was annotated as a full *Ds *element in maizesequence.org. Among the 44 *Ds-l3 *elements, 42 were partially annotated as *Ds *and 2 are new. Notably, of the 486 *Ds-l4 *elements, only 62 had been partially annotated as *Ds *elements, while the remaining 424 had been missed. Despite their dissimilarities with the *Ac *sequence, the *Ds-l4 *elements themselves are related to each other. A Jalview phylogram [35,36] shows that *Ds-l4 *elements fall into 3 main clusters, after excluding from the alignment the 30 elements that are larger than 5 kb (Additional File 2, Figure S1). The *Ds-l4 *elements in each cluster have a high degree of similarity throughout. Cluster 1 (top part of the phylogenetic tree) contains 57 elements, cluster 2 (middle of the phylogenetic tree) contains 339 elements, and cluster 3 (bottom of the phylogenetic tree) contains 60 elements.

### Nature of *Ds *insertion sites

*Ds1 *and *Ds2 *sequences have been shown to cause mutations when they insert into genes [6,12,30,31,37-39], so it is not surprising that several *Ds *and *Ds-l *sequences were found in or near genes. As shown in Table 3, 37 *Ds1 *(11%), 5 *Ds2 *(13%), 2 *Ds-l3 *(5%), and 34 *Ds-l4 *(7%) elements had inserted in or within 200 bp of a gene model. Given the propensity for *Ac *and *Ds *to insert into or near genes, it was surprising that relatively few *Ds *elements mapped into or near genes in B73. It would appear, then, that somewhat lower percentages of *Ds-l *than *Ds *elements are found near genes. Similarly, slightly higher percentages of *Ds-l *elements are inserted in repetitive or intergenic low-copy DNA (49.8% vs. 41.5%, respectively). Overall higher percentages of *Ds*-related elements are found in repetitive DNA sequences than would have been expected from prior genetic analyses of recently transposed *Ac *or *Ds *elements in maize [15,16].

**Table 3:** Nature of sequences adjacent to *Ds *insertions in B73

Element	Genes (in or within 200 bp)	Repetitive DNA	Intergenic DNA	Total
*Ds1*	37	155	139	331
*Ds2*	5	13	21	39
*Ds-l3*	2	25	17	44
*Ds-l4*	34	180	272	486
*Ac-like*	0	2	1	3

Totals	78	375	450	903

### *Ds *and *Ds-like *elements carrying gene fragments

**Figure 3:**
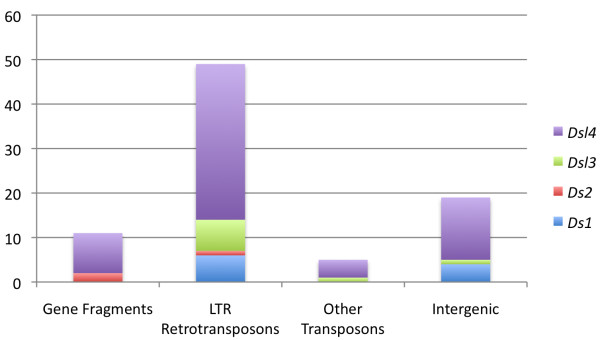
**DNA sequences inserted within *Ds *elements**. The sequences can originate from genes, LTR retrotransposons, other DNA transposons, or intergenic DNA.

Although transposons from other superfamilies, such as MULEs [40], CACTA [41], and *Helitrons *[42,43], can take up gene fragments, this property has not been reported for members of the *hAT *superfamily, to which *Ds *belongs [7]. In this study, we have identified both *Ds2 *and *Ds-l4 *elements that carry gene fragments (Figure 3).

**Figure 4:**
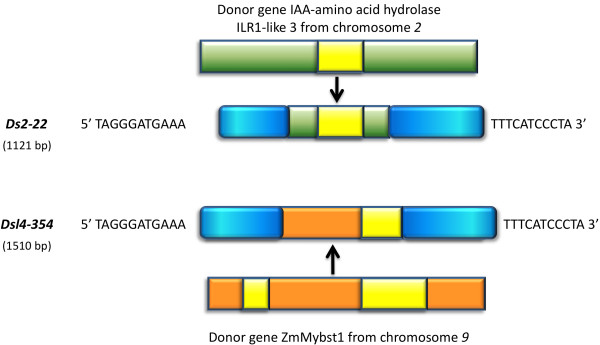
**Genic DNA capture by *Ds *elements**. *Ds2-22 *and *Ds-l4-354 *are located on chromosomes 9 and 8, and their inserted gene fragments, containing intron sequences, are from chromosome 2 and 9, respectively. Exons are diagrammed in green or orange, introns in yellow, and *Ds *transposon sequences in blue.

Two *Ds2 *elements appear to have captured gene fragments. *Ds2-22 *carries exons 5 and 6 and the intervening intron from an IAA amino acid hydrolase *ILR1-like3 *gene. This gene fragment appears to have been copied from its original location on chromosome 2 onto *Ds2-22*, which resides on chromosome 9 of B73 (Figure 4). The sequence carried in the *Ds2-18 *element is annotated as a complete gene encoding hypothetical protein LOC100383699. This annotation is supported by EST and mRNA evidence. However, all copies of this coding sequence are transposon-borne in B73, raising doubts as to the functional significance of the transcript in the sequence database.

**Figure 5:**
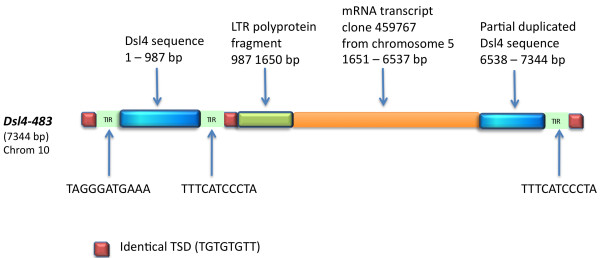
**Compound *Ds-l4 *element with trapped extraneous sequences between its termini**. The *Ds-l4-483 *element on chromosome 10 is 7344-bp in length. Bases 1-987 of Ds-l4-483 correspond to a regular *Ds-l4 *element with perfect TIRs flanked by a TSD. Following this are a fragment of an LTR-retrotransposon polyprotein (988 to 1650), a transcribed sequence from chromosome 5 (1651 to 6537), and a duplicate, but truncated, copy of the first *Ds-l4 *in this compound element, ending in a perfect TIR and an additional copy of the TSD (6538 to 7344).

Similarly, some *Ds-l4 *elements appear to have captured gene fragments. *Ds-l4-354*, which resides on chromosome 8 of B73, carries a fragment of the *ZmMybst1 *gene, which is located on chromosome 9 (Figure 4). The 7344-bp *Ds-l4-483 *element on chromosome 10 is an example of a compound *Ds-l4 *element that has trapped extraneous sequences between its termini (Figure 5). Bases 1-987 of *Ds-l4-483 *correspond to a regular *Ds-l4 *element with perfect TIRs flanked by a TSD. Directly following this are: a fragment of an LTR-retrotransposon polyprotein (988 to 1650), a transcribed sequence from chromosome 5 (1651 to 6537), and a duplicate, but truncated, copy of the first *Ds-l4 *in this compound element, ending in a perfect TIR and an additional copy of the TSD (6538 to 7344).

Two additional sets of transcribed sequences, found in the GenBank mRNA database, are carried by repetitive *Ds-l4 *elements. *Ds-l4-93*, *Ds-l4-135*, *Ds-l4-307*, and *Ds-l4-343 *contain the two putative exons of *Zea mays *clone 1443778 mRNA and *Ds-l4-244*, *Ds-l4-275*, and *Ds-l4-368 *contain the two putative exons of *Zea mays *clone 1451145 mRNA. The introns separating these exons in the elements end in canonical GT-AG. Neither mRNA encodes a protein with a homolog in the sequence databases, so again, it is conceivable that the only copies of the transcribed sequences in the genome are those carried in the transposons. Nevertheless, the presence in different chromosomes of multiple copies of two sets of two *Ds-l4 *elements with almost identical transcribed sequences suggests that these elements transposed in the recent past, after acquisition of the sequences.

### Nongenic insertions in *Ds *element

Large insertions were found in all *Ds *element classes (Figure 3). Not surprisingly, the majority were LTR retrotransposons, which constitute the bulk of the maize genome [44]. Ten *Ds1 *elements were much longer than average, measuring between 0.8 and 24 kb. LTR retrotransposons were inserted in six of them (Additional File 1, Table S2). Although the average length of *Ds2 *elements was approximately 1.4 kb, some, such as *Ds2-13*, were as long as 12.7 kb as a result of LTR retrotransposon insertions (Additional File 1, Table S3). Similarly, LTR retrotransposons were inserted in 7 *Ds-l3 *and 35 *Ds-l4 *elements (Additional File 1, Tables S4 and S5). Though less numerous, DNA transposons were also found inside of *Ds *elements. For example, a *Ds-l4 *element was nested inside of *Ds-l3-15*. This nesting pattern is common for LTR retrotransposons [45], but rarer for *Ds *transposon types, probably because nested *Ds *elements acquire chromosome-breaking properties [46] and would be selected against. In agreement, no chromosome-breaking double *Ds *[5] was detected in B73. In addition to transposons, intergenic sequences without obvious transposons properties could be found inside of *Ds *elements. These most likely represent DNA capture events that do not include coding sequences. Intergenic DNA sequences of various lengths were found in 4 *Ds1*, 1 *Ds-l3*, and 14 *Ds-l4 *elements (Additional File 1, Tables S1, S3, and S4).

### Mobility of *Ds *and *Ds-l *elements

The terminal 200 bp at either end of *Ac *are required for wild-type levels of transposition [47]. Within these terminal sequences, there are multiple AAACGG repeats that bind to the *Ac *transposase in a cooperative fashion and are, most likely, the subterminal sequences that impart specificity to the transposition reaction [8,22,23]. Only three copies of these hexamers have been shown to be required for transposition [23]. The number of B73 *Ds*-related elements with 3 or more copies of the subterminal repeat in each class are: 17 of 331 *Ds1*, all 39 *Ds2*, 37 of 44 *Ds-l3*, and 438 of 486 *Ds-l4 *(Table 4). Thus, the vast majority of *Ds2*, *Ds-l3*, and *Ds-l4 *elements contain 3 or more AAACGG repeats within their subterminal regions, but only a few *Ds1 *elements do. However, the lack of these hexamers does not preclude transposability, as some *Ds1 *sequences lack them and are still able to transpose, probably because the transposase can interact with sequences related to, though not identical with, the hexameric repeat [8].

**Table 4:** *Ds*- and *Ds-like *sequence with 3 or more copies of the hexamer sequence AAACGG within their subterminal regions

Chromosome	*Ds1*	*Ds2*	*Ds-l3*	*Ds-l4*	*Ac-Like*	Totals
1	3	7	3	76	1	90
2	0	5	3	42	1	51
3	0	2	6	48	0	56
4	4	2	5	63	0	74
5	5	4	3	42	1	55
6	0	0	3	30	0	33
7	2	3	5	37	0	47
8	1	6	5	38	0	50
9	1	2	0	24	0	27
10	0	4	3	32	0	39
Unknown	1	4	1	6	0	12

Totals	17	39	37	438	3	534

**Figure 6:**
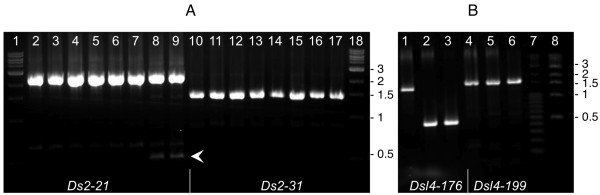
**PCR assay for *Ac*-driven excision of *Ds2 *and *Ds-l4 *elements**. (A) *Ds2-21 *and *Ds2-31 *elements. Lanes: 1 and 18, 1-kb ladder; 2 and 10, W22 inbred (*Wx*); 3 and 11, B73, pooled seedlings; 4 and 12, B73 plant #1; 5 and 13, B73 plant #2; 6 and 14, *wx-m7 *plant # 1; 7 and 15, *wx-m7 *plant #2; 8 and 16, F1 (B73-1 × *wx-m7*-2); 9 and 17, F1 (B73-2 × *wx-m7*-2). Only the *Ac*/+ heterozygous F1 individuals (lanes 8-9) showed the band expected from somatic *Ds2-21 *excision. The absence of that band in the *wx-m7(Ac) *homozygotes (lanes 6-7) can be attributed to the well-established negative dosage effect of *Ac *[59]. No somatic excision of *Ds2-31 *could be detected in the *Ac*/+ heterozygous F1 individuals (lanes 16-17). (B) *Ds-l4-176 *and *Ds-l4-199 *elements. Lanes: 1, B73, plant #1; 2, *wx-m7 *plant #2; 3, F1 (B73-1 × *wx-m7*-2); 4, B73, plant #1; 5, *wx-m7 *plant #2; 6, F1 (B73-1 × *wx-m7*-2); 7, 100-bp ladder; 8, 1-kb ladder. *Ds-l4*-176 is polymorphic, i.e., not shared between B73 and W22, and cannot be assayed. No somatic excision of *Ds-l4-199 *could be detected in the *Ac*/+ heterozygous F1 individuals (lane 6).

**Figure 7:**
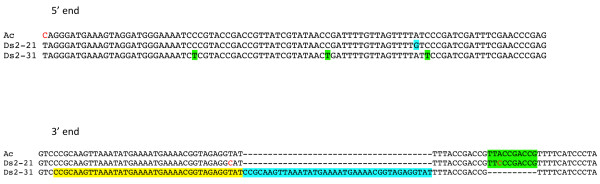
**Alignment of the 5' and 3' terminal 80 bp from *Ac, Ds2-21*, and *Ds2-31***. All three 5' ends align well, except for two SNPs in *Ds2-21*. In contrast, the 3'ends of *Ac *and *Ds2-21 *align well, but the 3' end of *Ds2-31 *is interrupted by a duplication and a deletion.

In order to test the mobility of the newly identified *Ds-l3*, *Ds-l4*, and control *Ds2 *elements in response to *Ac*, we developed the following PCR excision assay. We first identified *Ds-l3*, *Ds-l4*, and *Ds2 *elements in B73 that had inserted in single-copy DNA and were shared with the inbred W22. We then crossed B73 with the W22 genetic stock *c wx-m7(Ac) *and monitored filial and parental DNAs for the presence of a somatic *Ds *excision or "empty" site in the F1, but not in B73. Figure 6 shows the PCR results for representative elements. *Ds2-21 *shows a clear excision band in the F1, whereas *Ds2-31 *does not (Figure 6A), indicating that the former can transpose in response to *Ac*, but the latter cannot. An alignment of the 5' and 3' ends of *Ds2-21 *and *Ds2-31 *with *Ac *(Figure 7) reveals that *Ds2-31 *has undergone a duplication-deletion at the 3' end, which probably interferes with its mobility. On the other hand, none of the shared *Ds-l *elements tested (*Ds-l3-28*, *Ds-l4-118*, *Ds-l4-169*, *Ds-l4-199*, *Ds-l4-266*, *Ds-l4-337*, and *Ds-l4-378*) produced an excision band in the F1 (Figure 6B and data not shown). Thus, in spite of possessing subterminal AAACGG hexamers, neither *Ds-l3 *nor *Ds-l4 *elements are able to transpose. Possibly, the 5' and 3' ends of *Ds-l3 *and *Ds-l4 *elements, as well as of 17 of 39 *Ds2 *elements (alignment data not shown), are too defective to be mobilized by *Ac*.

### Sampling the *Ds *Elements of W22

The only complete maize genome sequence in the public domain is that of the B73 inbred. This genome is constantly being revised and recompiled, and serves as the *de facto *maize database for BLAST. The W22 inbred line is being utilized in genetic studies aimed at developing *Ac*/*Ds *transposon tagging resources [15,16], so knowledge of the full complement of *Ds-*related elements in W22 would be valuable. In the course of this study, we found that 9 of 18 *Ds *or *Ds-l *elements present in B73 were absent in W22 (e.g., *Ds-l4-176 *in Figure 6B), which precluded a PCR test for excision in the F1. To estimate the fraction of *Ds *insertion sites in W22 that are not shared with B73, we proceeded to isolate *Ds *5' junction sequences in W22 by a modified transposon display procedure, sequenced a set of random clones, identified 25 different junctions, and BLASTed them against the B73 genome. Only 13 matched *Ds *insertion sites in B73, (e.g., *Ds1-208*, *Ds2-35*, *Ds-l3-21*, *Ds-l4-61*, *Ds-l4-169*, and *Ds-l4-346*). Although only a small sample of *Ds *sites was analyzed, it is clear that a sizable fraction of *Ds *element insertion sites are polymorphic between these two inbred lines.

## Discussion

### Novel *Ds*-like elements

We report here a comprehensive analysis of *Ds *and *Ds-like *elements in the genome of B73, a maize inbred that lacks *Ac *activity (Additional File 1, Tables S2-S5). Two novel classes of *Ds*-like elements, *Ds-l3 *and *Ds-l4*, were identified in this study. Although they share TIR sequences with *Ac*, *Ds-l3 *elements differ greatly from *Ac *in the subterminal regions. Most *Ds-l3 *elements contain sequences from exons 2 and 3, the two largest exons of the 807-amino acid *Ac *transposase, but lack the C terminus, which is highly conserved among *hAT *elements and correlates with transposase activity [48]. The *Ac*-matching areas of these *Ds-l3 *elements have been incorrectly annotated as belonging to *Ac *elements in the maizesequence.org database.

*Ds-l4 *elements have the least in common with *Ac*, just 30 bp at either end, which explains why they had escaped detection until the present work, even though they comprise the majority of *Ds*-related sequences in the genome (54%). *Ds1 *elements, an established mobile clade within the *Ac*/*Ds *family, also share little in common with *Ac *beyond their TIR sequences. The main differences between *Ds-l4 *and *Ds1 *sequences are: their length, the former being about twice as large; the fraction of elements with 3 or more copies of the AAACGG subterminal repeat (438/486 vs. 17/331, respectively), and the nature of the non-*Ac *filler sequence. *Ds1 *elements share a common filler which exhibits minimal sequence variation [27]. *Ds-l4 *elements, on the other hand, have variable filler sequences, although their phylogenetic analysis reveals clusters of related elements (Additional File 2, Figure S1). The high copy number and high degree of similarity of the filler sequences in each cluster make it difficult to identify the original source of these sequences in the genome.

Our algorithm provided for up to 2-bp variation in either the element's TIR or the host TSD. To determine if the algorithm had missed any elements not flanked by a TSD, which is dispensable for transposition [49,50], we removed the TSD requirement from our script. The new search produced only 11 additional putative elements, with flanking host sequence identity ranging from 1 in 8 to 5 in 8. There were 3 *Ds1*, 1 *Ds2*, 2 *Dsl3*, and 5 not clearly related to any of the classes identified in this study. The ends of these 5 elements are well conserved, so probably all of them represent *Ds *elements at various stages of decay. This exercise suggests that we have essentially defined the entire *Ac-Ds *family in maize.

### Transposition of *Ds *and *Ds-l *elements

The hexameric repeat sequence AAACGG located in the STR of *Ac *is necessary for transposition [8,22]. All *Ds2*, 37 *Ds-l3*, and 438 *Ds-l4 *elements possessed hexamers in numbers judged sufficient for transposition [23]. However, none of the *Ds-l *elements tested by our PCR assay showed evidence of somatic excision in the presence of *Ac*, whereas the tested *Ds2 *elements did. The most likely explanation for this negative result is that the 5' and 3' ends of *Ds-l3 *and *Ds-l4 *elements are too defective to be mobilized by *Ac*. Alternatively, these elements may reside in hypermethylated DNA, which has been shown to inhibit Ac transposition [51]. The methylation status of *Ds *sequences was not analyzed, as this was beyond the scope of the present study. Multiple copies of the hexameric repeat may not be the key requirement for *Ds1 *mobility because some known mobile *Ds1 *elements contain just one copy of the repeat [11,27]. Of the 331 *Ds1 *elements in the B73 genome, 314 lack them. Kunze and Starlinger [8] have emphasized that the lack of these hexamers does not exclude an element from being able to transpose. *Ds1 *elements are presumed to be transposable because other subterminal sequences may also interact with the transposase complex.

The occurrence of LTR retrotransposon sequences in *Ds *and *Ds-like *elements is not surprising because these sequences constitute more than three quarters of the maize genome [18]. An answer to the question of whether or not their increased size renders these elements nontransposable can be sought in the existing literature. There are several examples of *Ds *elements with large, unrelated stretches of DNA that can still transpose, provided an *Ac *element is present in the genome [52-54]. More recently, a macrotransposon with a *Ds *element at one end and an *Ac *element at the other was shown to mobilize as much as 100 kb of intervening DNA consisting mostly of nested retrotransposons [55]. Thus, it is unlikely that increased size alone makes *Ds-l *elements incapable of tranposition.

### Gene capture

Several different types of DNA transposons, including the recently discovered *Helitrons*, are known to pick up fragments of genes from the genome. However, there are no reports of gene fragment capture by *hAT *elements in the literature. Here, we identified 2 *Ds2 *and 9 *Ds-l4 *elements containing gene fragments. How excisive DNA transposons acquire gene fragments is not known, but it has been suggested that host sequence capture may result from DNA replication errors during repair of the double strand breaks caused by transposon excision [56].

The notion that gene capture proceeds via genomic DNA, as in all other DNA transposons, is supported by the two cases of *Ds2 *elements with trapped gene sequences. In the *Ds2-22 *element, which carries a fragment of an IAA amino acid hydrolase ILR1-like 3 gene, the intron sequence between exons 5 and 6 is present, arguing against capture from an mRNA. *Ds2*-22 is on chromosome 9, whereas the donor gene is on chromosome 2 (Figure 4.). The second *Ds2 *element is more intriguing. A homologous transcript of unknown function, with putative start and stop codons, can be found in the maize mRNA sequence database. *Ds-l4 *elements can also carry database-supported, transcribed genomic sequences containing introns. For some of them, the donor gene can be identified. An example of a captured sequence from a known gene is provided by *Ds-l4-354*, with the element on chromosome 8 and the donor gene on chromosome 9 (Figure 4). However, putative donor gene sequences for other fragments could not be found in the current version of the B73 genome, suggesting that the corresponding *Ds-l4 *transcripts in the databases may represent nonfunctional transcriptional baggage.

### *Ds *content of different inbreds

The *Ds *content of the inbred W22, used in its *wx-m7 *version as the *Ac *source for the *Ds-l4 *excision assay, was surveyed in this study. Of 25 *Ds *or *Ds-l *junctions isolated from W22, only one-half (13) were shared by B73. Though these two inbreds fall into different heterotic groups [57], they represent germplasm adapted to the U.S. Corn Belt. We infer that, as has been found for retrotransposons [58], the content of most DNA transposons will be highly polymorphic among maize lines.

We can also compare the distribution of *Ds *elements accumulated over time in B73 with that of recently transposed *Ds *elements in W22, the inbred used by Vollbrecht et al. [15] in their studies. Additional File 3, Figure S2 shows the distribution of *Ds *elements in each pseudomolecule of the B73 genome. While a statistical comparison is unwarranted because of the smaller number of elements in our study (no chromosomal region has ≥10 insertions), a visual comparison reveals similar trends: low concentration of elements in the centromeric regions and high concentration of elements in the distal tips of some arms. particularly *1L, 2S, 5L, 7L*, and *8L*.

## Conclusion

Transposons, recognized today as ubiquitous components of eukaryotic genomes, were first identified six decades ago by their disruption of chromosomal integrity and their mutagenic effects on genes. The elegant genetic analysis of gene mutations that served as reporters for their movement served to elucidate many transposon properties, but the complexity of transposon families has only been revealed by whole genome sequencing projects. Here we report on an in-depth analysis of all the sequences in the maize genome that are related to *Dissociation *(*Ds*), the first transposon ever described. Our bioinformatic approach has uncovered over 900 *Ds*-related sequences, including two novel classes that account for the majority of elements in the family. These novel elements have diverged significantly from *Ds *and, though clearly related to *Ds*, do not appear to transpose in today's maize genome. Unlike actively transposing elements, many of the *Ds*-related elements are inserted in repetitive DNA, where they probably become immobile and begin to decay. A second maize inbred line shared only half of its *Ds *insertion sites with B73, suggesting that present-day maize inbred lines differ greatly in their DNA transposon make-up.

## Authors' contributions

Conceived and designed the experiments: CD and HKD. Performed the experiments: AH, LH, JC. Analyzed the data: CD, AH, LH, JC, HKD. Wrote the paper: CD and HKD. All authors read and approved the final manuscript.

## Supplementary Material

Additional file 1 **Supplemental tables**. Table S1. PCR primers used to assay *Ds *and *Ds-l *somatic excisions. Table S2. Description of *Ds1 *elements. Table S3. Description of *Ds2 *elements. Table S4. Description of *Ds3 *elements. Table S5. Description of *Ds4 *elements. Click here for file

Additional file 2 **Figure S1**. - Phylogenetic tree of 456 *Ds-l4 *elements. All *Ds-l4 *elements other than the 30 containing insertions of known transposons, were aligned by the high-speed multiple sequence alignment program MAFFT (Multiple Alignment using Fast Fourier Transform) http://www.ebi.ac.uk/Tools/msa/mafft/. The phylogenetic tree with the shortest branch lengths was built by Jalview software using the neighbor joining algorithm. Three major clusters of filler sequences are identified. There are 57 elements in cluster 1 which is the top part of the phylogenetic tree and 60 elements in cluster 3, the bottom part of the tree. These clusters are relatively more divergent than cluster 2, which is the largest one with 339 elements in the middle of the phylogenetic tree. Click here for file

Additional file 3 **Figure S2**. Distribution of *Ds *elements in each of the 10 B73 pseudomolecules. The X axis shows the length of each chromosome in megabases (Mb) and the y axis shows the number of *Ds *insertions in each 5-Mb bin. Approximate centromere positions are indicated with a black circle. Click here for file
