# Supplementary figures and images for: The complete Ac/Ds transposon family of maize
Source: BMC Genomics. 2011 Dec 1;12:588. doi: 10.1186/1471-2164-12-588 (PMC3260210; doi:10.1186/1471-2164-12-588)

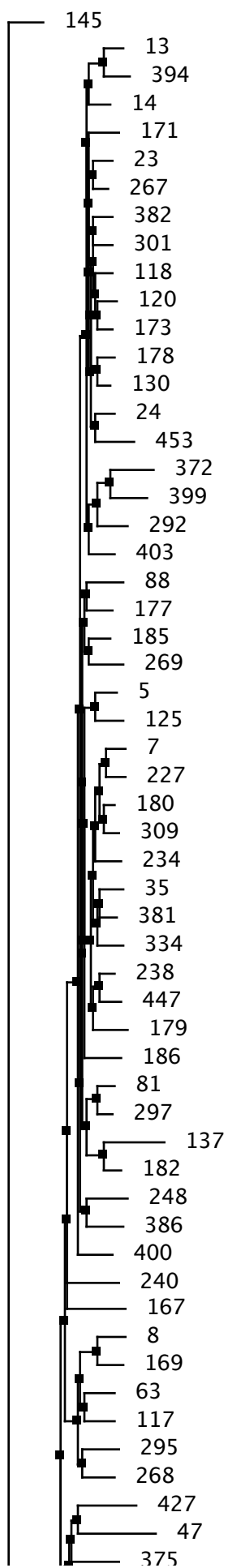

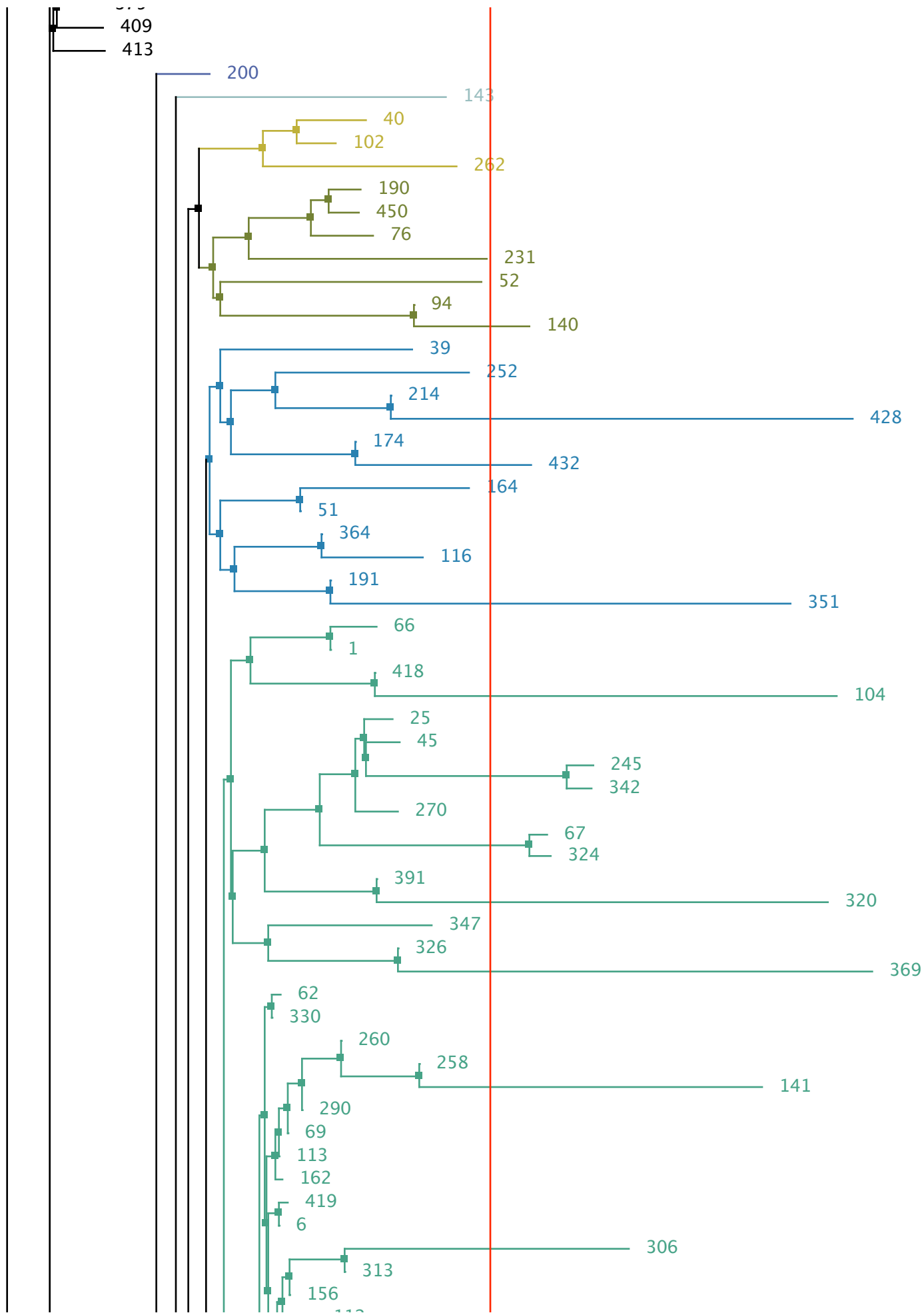

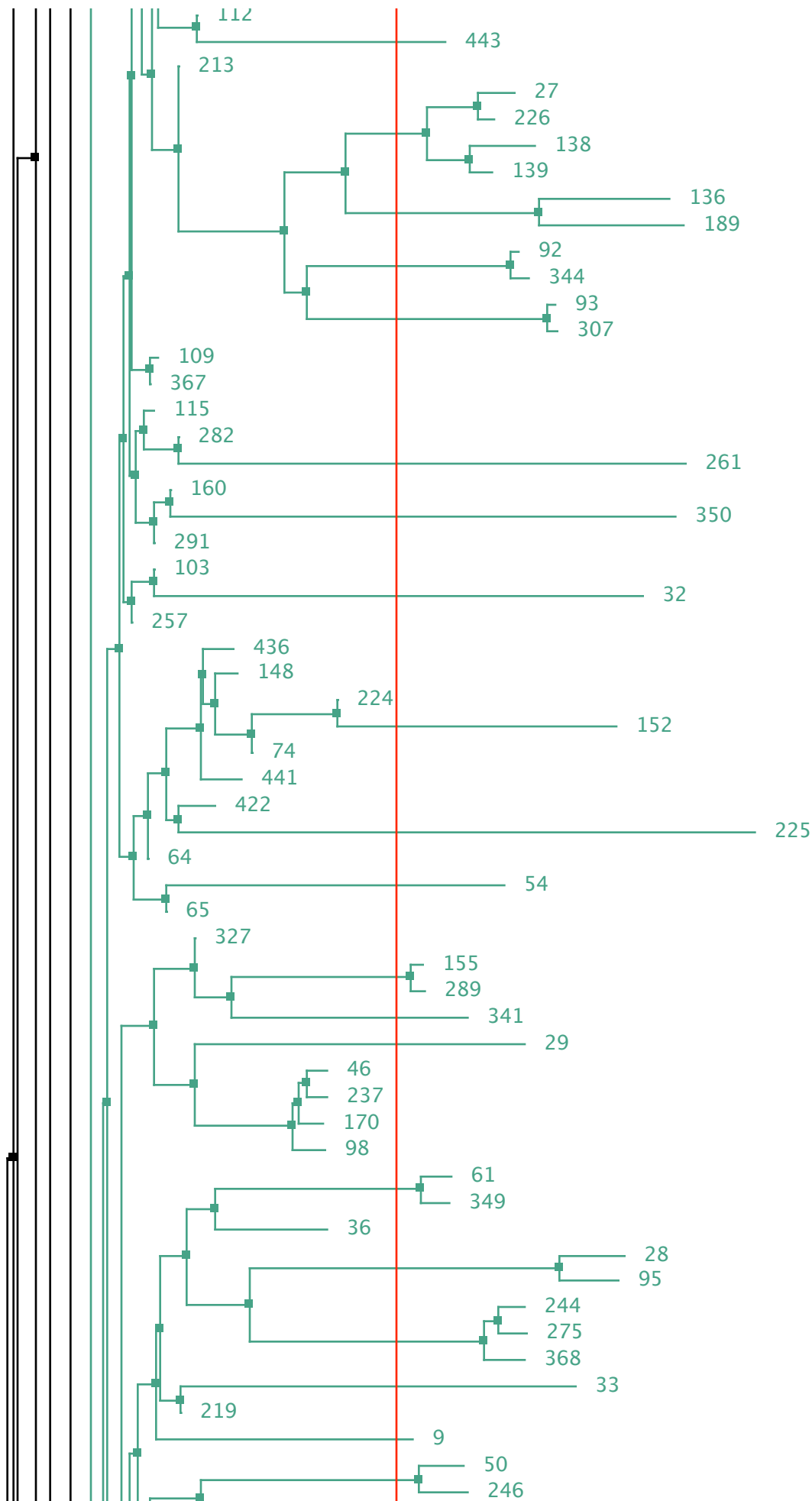

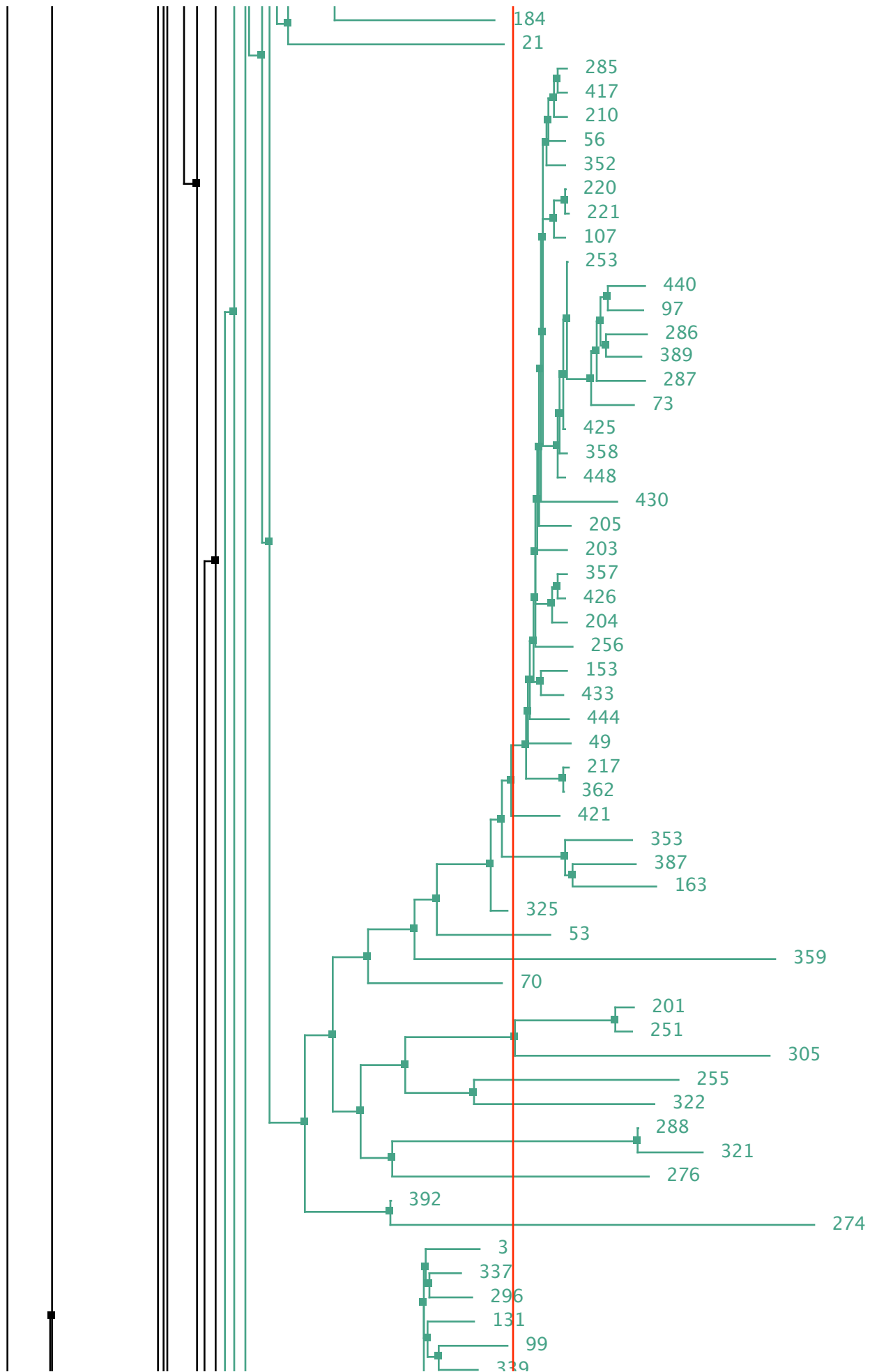

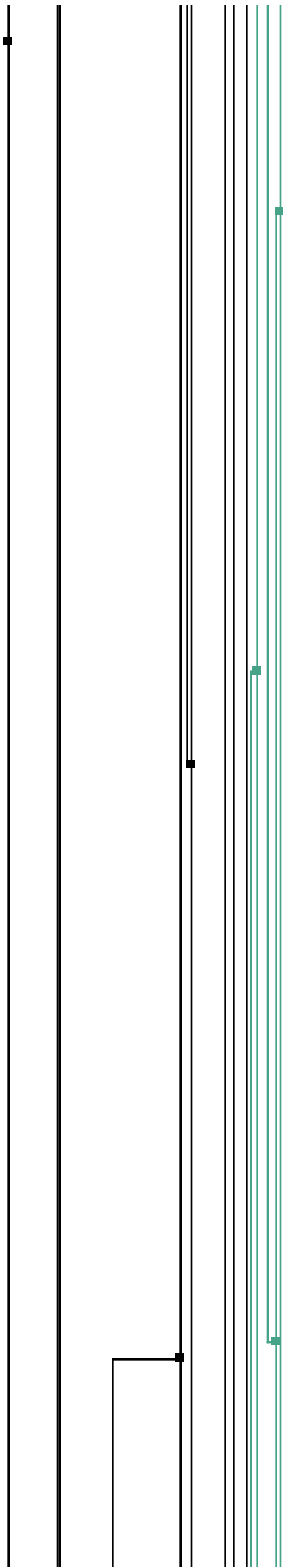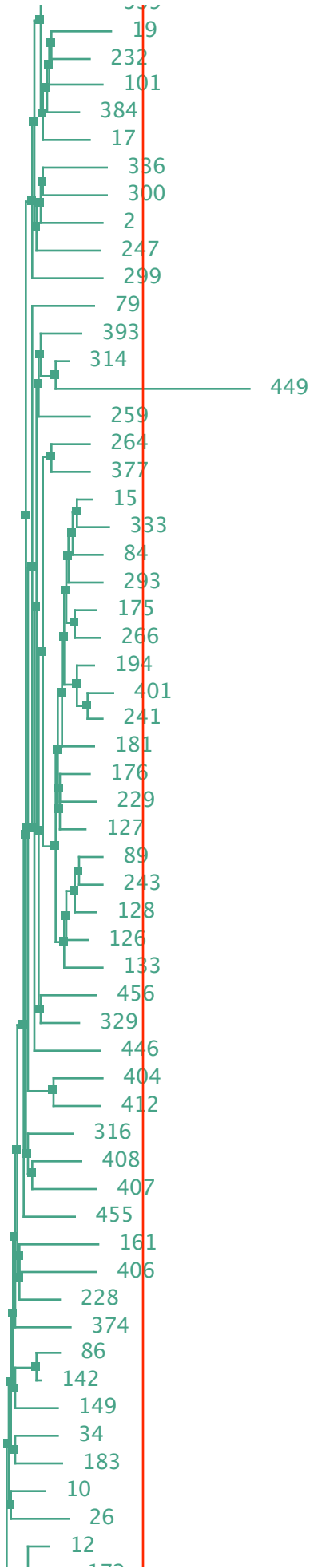

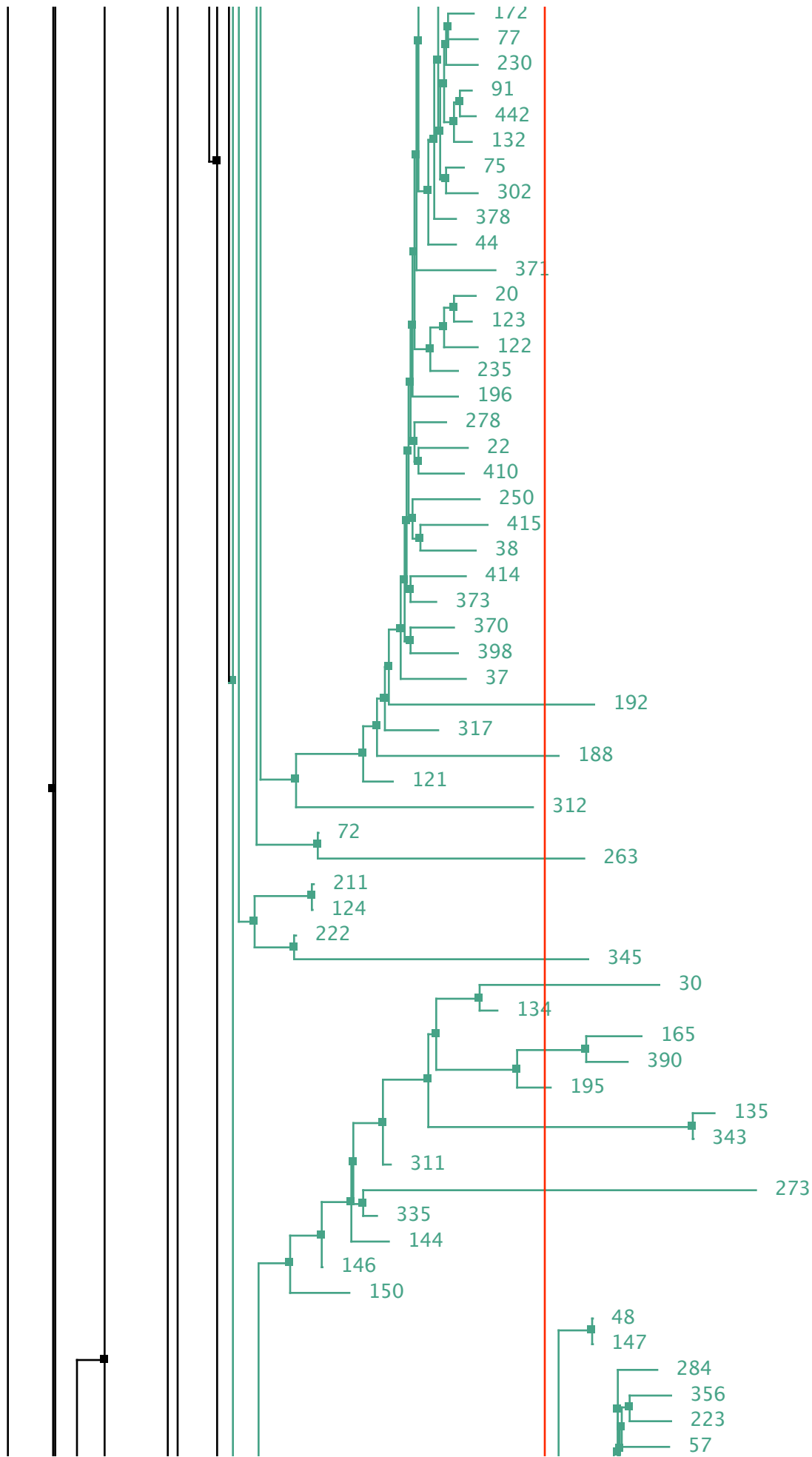

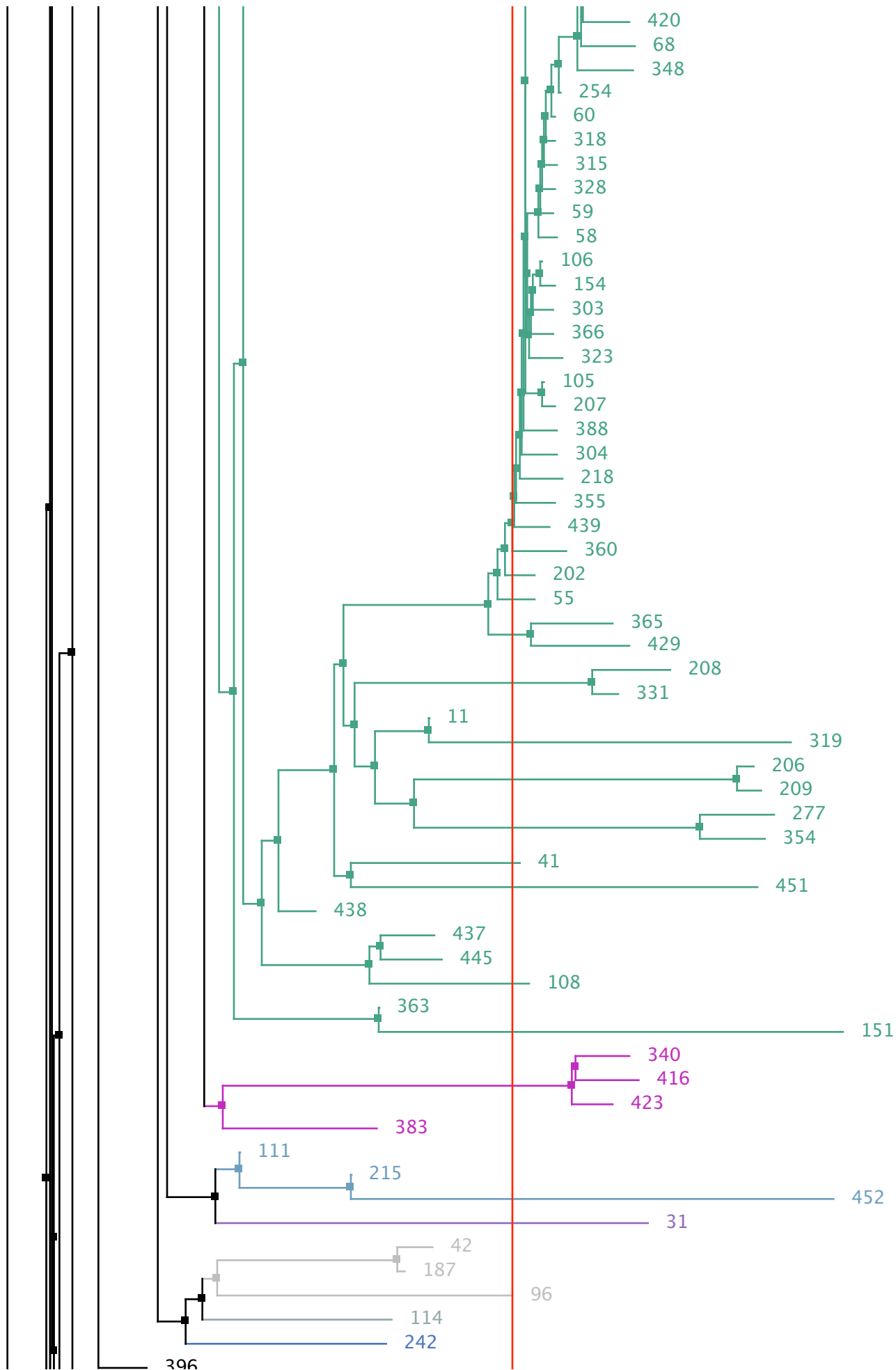

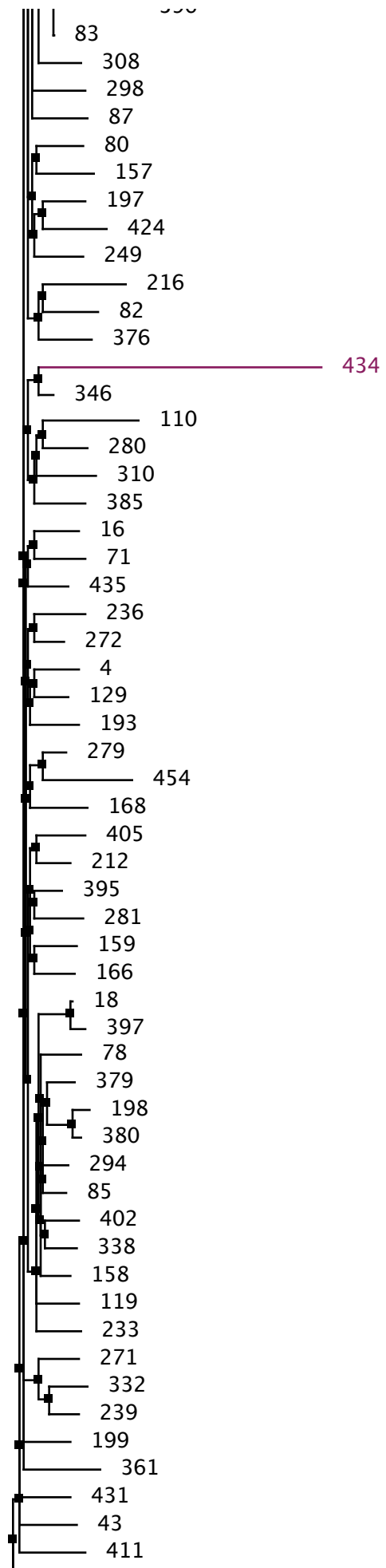

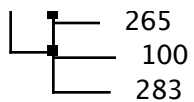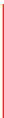

Supplement: Additional file 2 — Figure S1. - Phylogenetic tree of 456 Ds-l4 elements. All Ds-l4 elements other than the 30 containing insertions of known transposons, were aligned by the high-speed multiple sequence alignment program MAFFT (Multiple Alignment using Fast Fourier Transform) http://www.ebi.ac.uk/Tools/msa/mafft/. The phylogenetic tree with the shortest branch lengths was built by Jalview software using the neighbor joining algorithm. Three major clusters of filler sequences are identified. There are 57 elements in cluster 1 which is the top part of the phylogenetic tree and 60 elements in cluster 3, the bottom part of the tree. These clusters are relatively more divergent than cluster 2, which is the largest one with 339 elements in the middle of the phylogenetic tree. [file 1471-2164-12-588-S2.PDF]
