# Supplementary material for: The complete Ac/Ds transposon family of maize
Source: BMC Genomics. 2011 Dec 1;12:588. doi: 10.1186/1471-2164-12-588 (PMC3260210; doi:10.1186/1471-2164-12-588)
Supplement: Additional file 3 — Figure S2. Distribution of Ds elements in each of the 10 B73 pseudomolecules. The X axis shows the length of each chromosome in megabases (Mb) and the y axis shows the number of Ds insertions in each 5-Mb bin. Approximate centromere positions are indicated with a black circle. [file 1471-2164-12-588-S3.PPT]

## Slide 1
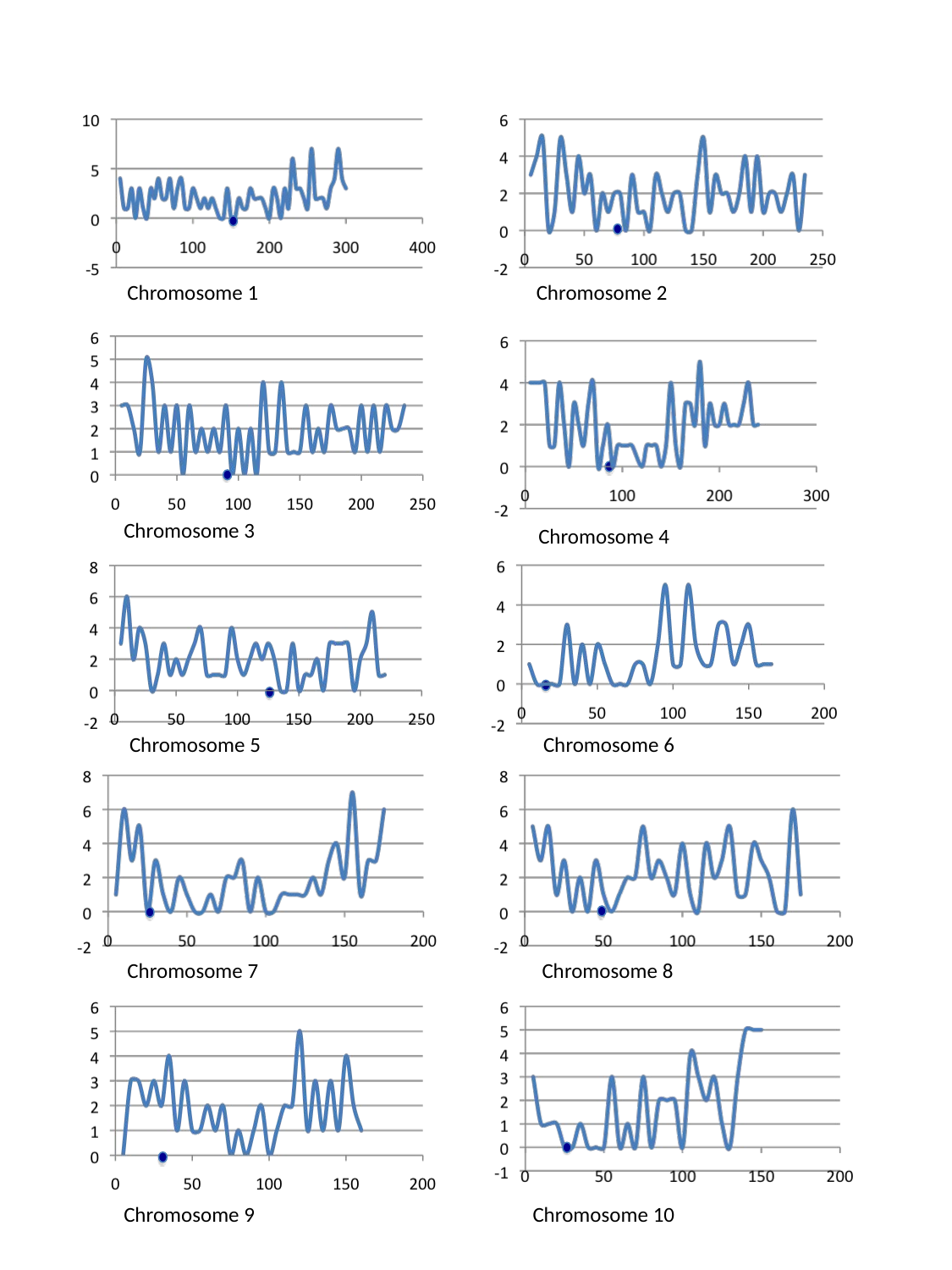

Chromosome 1
Chromosome 2
Chromosome 3
Chromosome 4
Chromosome 5
Chromosome 6
Chromosome 7
Chromosome 8
Chromosome 9
Chromosome 10
